# Insulin resistance (TyG index) and body mass index as metabolic biomarker combined with ApoE genotype to diagnose Alzheimer’s disease

**DOI:** 10.3389/fnagi.2026.1731547

**Published:** 2026-02-18

**Authors:** Renyu Chen, Shiyu Fan, Cihan Di, Hao Wu, Zhihong Shi, Feng Liu, Zhaoyang Lv, Shuai Liu, Yong Ji

**Affiliations:** 1 Huanhu Hospital Affiliated to Tianjin Medical University, Tianjin, China; 2 Tianjin Key Laboratory of Cerebrovascular and Neurodegenerative Diseases, Department of Neurology, Tianjin Huanhu Hospital, Tianjin Dementia Institute, Tianjin, China; 3 Anqiu City People’s Hospital, Shandong, China; 4 Tianjin University of Traditional Chinese Medicine, Tianjin, China; 5 The Second Affiliated Hospital of Qiqihar Medical College, Heilongjiang, China

**Keywords:** Alzheimer’s disease, APOE gene, body mass index, insulin resistance, TyG index

## Abstract

**Background:**

Growing evidence suggests that both ApoE genotype and metabolic disturbances including insulin resistance (IR) and obesity constitute risk factors for Alzheimer’s disease (AD). However, large-scale studies investigating whether ApoE genotype interacts with metabolic abnormalities to indirectly impair cognitive function in AD remain scarce.

**Objective:**

This cross-sectional study aimed to explore the associations between ApoE genotype, metabolic disturbances [IR assessed by triglyceride-glucose (TyG) index and body mass index (BMI)], and cognitive function in AD patients.

**Methods:**

We analyzed 1,162 clinically diagnosed probable AD patients from the Cognitive Impairment Clinic at Tianjin Huanhu Hospital. Participants were categorized by ApoE ε4 carrier status. Metabolic parameters were evaluated using the TyG index and BMI. Mediation effect models were employed to assess the relationships between ApoE genotype, metabolic indices, and cognitive function.

**Results:**

ApoE ε4 carriers exhibited significantly lower BMI (*P* < 0.001) and higher TyG index (*P* < 0.001) compared to non-ApoE ε4 carriers. Significant TyG index elevation in ApoE ε4 carriers was observed in AD patients with Mini-Mental State Examination (MMSE) > 20 (*P* = 0.0036) and MMSE 10–20 (*P* = 0.009). Mediation analysis revealed that ApoE ε4 exerted 73.4% of its negative effect on cognition through direct pathways, while 9.7 and 16.9% were mediated through BMI reduction and TyG elevation, respectively.

**Conclusion:**

ApoE ε4 carriers demonstrate a distinct metabolic profile characterized by lower BMI and elevated TyG index, associated with poorer cognitive performance. Our findings suggest that ApoE ε4 may indirectly influence AD cognition through metabolic pathways, highlighting early interventions targeting ApoE-related metabolic dysregulation as potential strategies to delay AD progression.

## 1 Introduction

Alzheimer’s disease (AD), a progressive neurodegenerative disorder, manifests clinically as gradual memory loss and cognitive decline (Khan et al., 2020). The widely recognized neuropathological hallmarks include extracellular β-amyloid (Aβ) deposition, intraneuronal neurofibrillary tangles composed of hyperphosphorylated tau protein, neuronal degeneration, and neuroinflammation (Wirth et al., 2013). With accelerating global population aging, AD has emerged as a critical public health challenge. Recent epidemiological data indicate a crude prevalence rate of 9.1% for dementia among Chinese individuals aged ≥ 65 years (Gan et al., 2024). Global dementia cases are projected to increase from 50 million in 2010 to 113 million by 2050 (Scheltens et al., 2021). According to the China Alzheimer’s Disease Report 2024, there were approximately 16.99 million prevalent cases of AD and other dementias in China in 2021, with AD-related deaths reaching 490,000—accounting for 25.2% of global dementia mortality (Wang et al., 2024). These statistics underscore the urgency of identifying modifiable risk factors and implementing early preventive strategies. While multiple etiological factors contribute to AD pathogenesis, mounting evidence suggests metabolic dysregulation may represent a crucial pathophysiological mechanism underlying AD development (de la Monte and Wands, 2008).

Apolipoprotein E (ApoE) gene is the most impactful genetic risk factor for AD. Despite the growing number of identified genetic risk factors, ApoE remains the strongest and most common, influencing over half of AD cases. Specifically, the ApoE ε4 is a major genetic risk factor for AD, increasing the risk in monozygotic twins by up to 15 times in a gene-dosage-dependent manner (Strittmatter et al., 1993). In contrast, ApoE ε2 nearly halves the risk of AD and is considered a protective factor against cognitive impairment (Corder et al., 1994). The ApoE ε4 has been definitively established as a primary genetic risk factor for AD (Corder et al., 1993). ApoE is primarily produced by peripheral and astrocytes in the central nervous system (CNS), playing a key role in cholesterol transport and regulating lipid transport and injury repair in the brain (Liu et al., 2013). Previous studies have shown that ApoE can regulate lipoprotein metabolism and also participate in the pathogenesis of AD by modulating brain insulin signaling, neuroinflammation, and synaptic plasticity (Mahley et al., 2006). Notably, peripheral metabolic abnormalities in ApoE ε4 carriers, such as hypertriglyceridemia and insulin resistance, may exacerbate the development of AD by increasing blood-brain barrier permeability, affecting intracerebral energy metabolism, and creating a vicious cycle of “metabolic-neuroinflammatory” (Cunnane et al., 2016).

IR and body BMI have long been recognized as core metrics for assessing metabolic health. IR primarily manifests as reduced insulin responsiveness in target tissues (Park et al., 2015), with common forms predominantly driven by energy intake-expenditure imbalance rather than genetic predisposition (Michailidis et al., 2022), predisposing individuals to obesity and type 2 diabetes (Sêdzikowska and Szablewski, 2021). Compared to the invasive hyperinsulinemic-euglycemic clamp method, the TyG index serves as a non-invasive and cost-effective alternative for IR screening. Notably, elevated TyG levels demonstrate strong correlations with accelerated cognitive decline in AD cohorts, suggesting its potential role in predicting disease progression (Tian et al., 2023). The relationship between BMI and neurodegenerative disorders exhibits a paradoxical pattern: midlife obesity (BMI ≥ 30 kg/m^2^) is significantly associated with increased dementia risk, while late-life weight loss may reflect disease-induced catabolic states, serving as a prodromal biomarker of neuropathological burden (Kivimäki et al., 2018).

While current research has advanced our understanding, critical gaps persist regarding the interaction dynamics between ApoE ε4 and metabolic disturbances (TyG index/BMI) in AD populations, particularly their specific cognitive consequences. This study systematically investigates the interplay of ApoE ε4 status with metabolic dysregulation in AD patients, with dual objectives to determine whether ApoE ε4-driven metabolic perturbations are causally linked to disease progression, and to establish a precision intervention framework for early metabolic-targeted strategies in genetically susceptible AD populations.

## 2 Materials and methods

### 2.1 Participants

This study involved a consecutive sample of participants recruited from the Cognitive Impairment Clinic in Huanhu Hospital Affiliated to Tianjin Medical University, China. This prospective study was approved by the Ethics Committee of Huanhu Hospital in Tianjin, and all procedures involving participants were conducted following the institution’s guidelines. All participants provided both oral and written informed consent. This study was conducted following the Helsinki Declaration clinical approval from the Ethical Redistribution, Tianjin Huanhu Hospital [(Jinhuan) Luncheon Review Nos. (2023-157) and (2024-175)]. Moreover, the study was registered on the Chinese Clinical Trial Registry website, bearing registration number ChiCTR2400080663.

### 2.2 Inclusion and exclusion criteria

#### 2.2.1 Inclusion criteria

Met international diagnostic standards for clinically probable AD according to the National Institute on Aging-Alzheimer’s Association (NIA-AA) working group criteria (McKhann et al., 2011). Age ≥ 18 years with preserved basic audiovisual function.

#### 2.2.2 Exclusion criteria

Secondary dementia diagnoses (e.g., vascular dementia, infectious encephalopathies, other neurodegenerative dementias); Concurrent severe metabolic disorders (end-stage renal disease, decompensated hepatic cirrhosis); Recent pharmacological interventions (glucocorticoids, statins, or immunosuppressants within 3 months prior to enrollment).

### 2.3 Standardized collection of the clinical data

During the same visit, all patients underwent an extensive standardized evaluation, following standard procedures, and blood collection. This assessment encompassed a standardized clinical, cognitive, behavioral, and functional protocol, including the Montreal Cognitive Assessment (MoCA) and the Clinical Dementia Rating (CDR) to stratify severity and monitor progression. The presence of neuropsychiatric symptoms was assessed using the Neuropsychiatric Inventory (NPI). Brain magnetic resonance imaging (MRI) scans were performed on all patients using either a 3 Tesla scanner to exclude cortical infarcts/hemorrhage or brain tumors. Vascular risk factors, comorbidities, and medication data were evaluated during the clinical assessment. Diabetes was defined as a fasting glucose greater than or equal to 126 mg/dL or the use of diabetes medications. Lifetime diagnosis of hypertension and dyslipidemia and use of antihypertension or hypolipidemic medications were determined by interview. Body mass index (BMI) was collected for all patients. TyG Index was calculated according to the following formula: TyG index = ln {[triglyceride (mg/dL) × glucose (mg/dL)]/2} (Sánchez-García et al., 2020).

### 2.4 ApoE genotyping

Genomic DNA was extracted from whole peripheral blood using the Maxwell^®^16 Instrument (Promega) and Maxwell^®^16 Blood DNA Purification Kit (Benussi et al., 2022). Next, PCR amplification of the ApoE rs429358 and rs7412 regions was performed using Promega’s GoTaq^®^ Hot-Start Polymerase. The PCR products were purified using 0.5 mL centrifugal filter from Merck Millipore’s Amicon^®^Ultra. Cycle sequencing was performed using the AB Prism BigDye Terminator Sequencing Kit 3.1 (Life Technologies), following the manufacturer’s guidelines. Subsequently, the obtained sequence was purified utilizing a MicroSEQ ID sequencing cleanup kit (Life Technologies) and loaded onto a 3,500 gene analyzer (Life Technologies). The sequences were analyzed using Chromas software (Technelysium Pty Ltd.).

### 2.5 Statistical analysis

Data was analyzed using SPSS 27.0. Continuous variables that conform to a normal distribution are expressed as mean ± standard deviation. The median and interquartile range represent continuous variables that do not follow a normal distribution. Clinical scores were analyzed by one-way analysis of variance followed by least significant differences tests. If clinical scores did not conform to normal distribution, they were analyzed using the Wilcoxon rank-sum test. Between-group differences in clinical features were assessed using the Kruskal-Wallis test and chi-square test for categorical variables, as appropriate. A mediating effect model was employed to investigate whether the TyG/BMI indices mediated the relationship between the ApoE gene and cognitive function. Statistical significance was set at *P* < 0.05 for all tests.

## 3 Results

### 3.1 Participant characteristics

The analysis included 1,162 patients with suspected AD, among whom 795 individuals (68.4%) underwent plasma ApoE genotyping (Table 1). Based on ApoE ε4 carrier status, participants were stratified into ApoE ε4 carriers (*n* = 343) and non-ApoE ε4 carriers (*n* = 452). Baseline demographic and clinical characteristics between groups are detailed in Table 1. The cohorts demonstrated comparable profiles in age, sex distribution, educational attainment, lifestyle factors, and medical history (all *P* > 0.05). Notably, ApoE ε4 carriers exhibited significantly lower Mini-Mental State Examination (MMSE) scores compared to non-carriers (*P* < 0.001), reinforcing the established association between ApoE ε4 and accelerated cognitive decline in AD progression.

**TABLE 1:** Basic information of ApoE ε4 and Non-ApoE ε4 patients.

Variant	ApoE ε 4 (*N* = 343)	Non-ApoE ε 4 (*N* = 452)	*P*
Age (years), [median (IQR)]	71 (12)	74 (11)	0.229
Sex, *n* (%)			0.097
Man	164 (47.8)	243 (53.8)	
Education, *y* [median (IQR)]	9 (3)	9 (6)	0.402
Smoking, *n* (%)	98 (28.6)	124 (27.4)	0.723
Alcohol, *n* (%)	89 (25.9)	117 (25.9)	0.984
Hypertension, *n* (%)	127 (37)	171 (37.8)	0.816
T2DM, *n* (%)	68 (19.8)	98 (20.8)	0.736
Heart disease, *n* (%)	37 (10.8)	47 (10.4)	0.860
Stroke, *n* (%)	29 (8.5)	37 (8.2)	0.892
MMSE score, [median (IQR)]	14 (8)	15 (11)	< 0.001***

IQR, interquartile range; MMSE, Mini-Mental State Exam; T2DM, type 2 diabetes mellitus; ***P < 0.001.

### 3.2 Association between ApoE genotype and metabolic markers: BMI and TyG index

**FIGURE 1:**
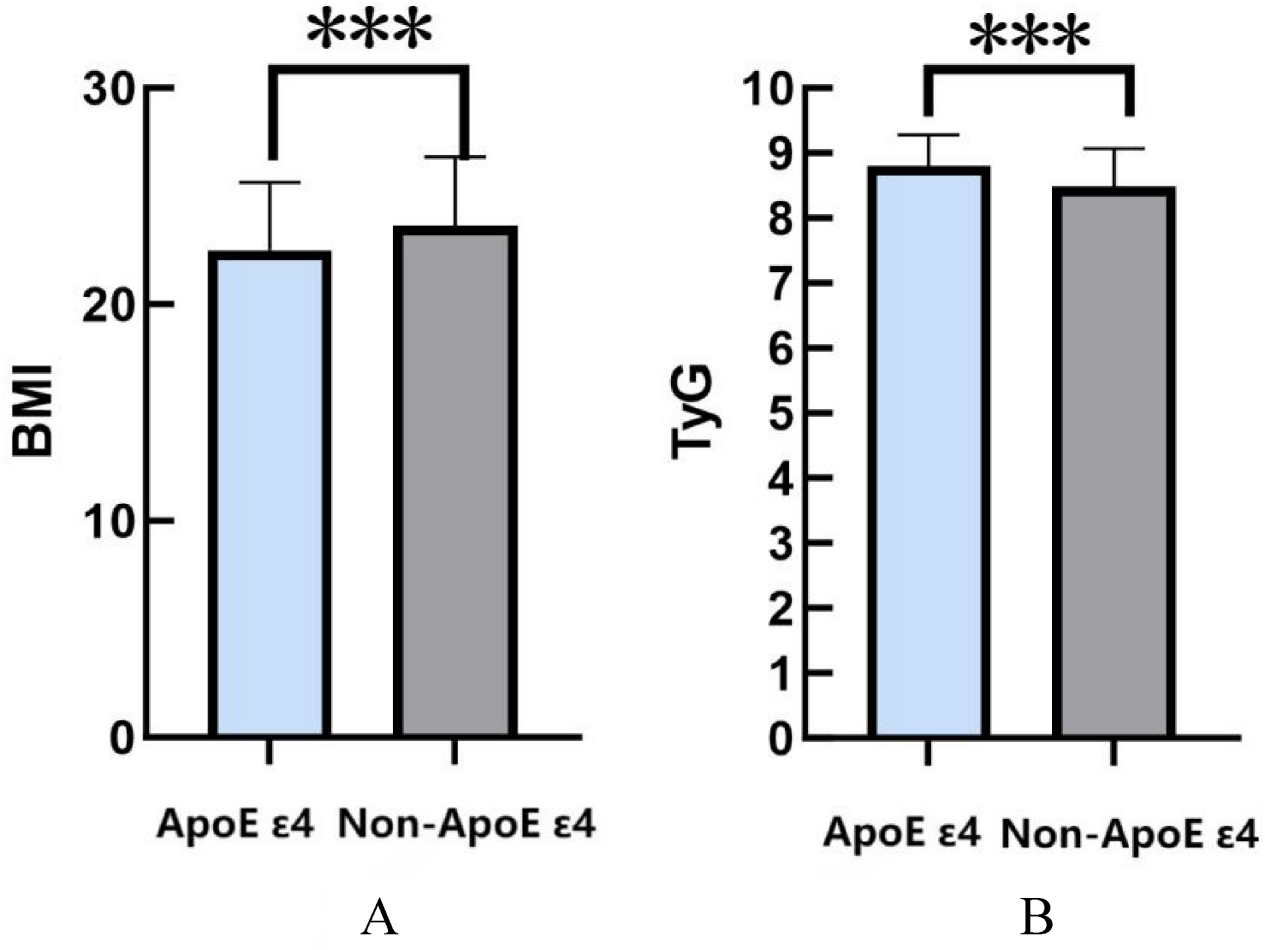
**(A)** The relationship between ApoE ε4 gene and BMI. **(B)** The relationship between ApoE ε4 gene and TyG. *P < 0.05; **P < 0.01; ***P < 0.001.

Using BMI and TyG index as primary evaluation metrics, this study investigated the relationship between ApoE ε4 carrier status and metabolic disturbances. The analysis revealed that ApoE ε4 carriers exhibited significantly lower BMI values compared with non-carriers (*P* < 0.001). Conversely, ApoE ε4 carriers demonstrated markedly elevated TyG indices relative to their non-carrier counterparts (*P* < 0.001) (Figure 1).

### 3.3 Gender-specific associations between ApoE genotype and metabolic profiles

**FIGURE 2:**
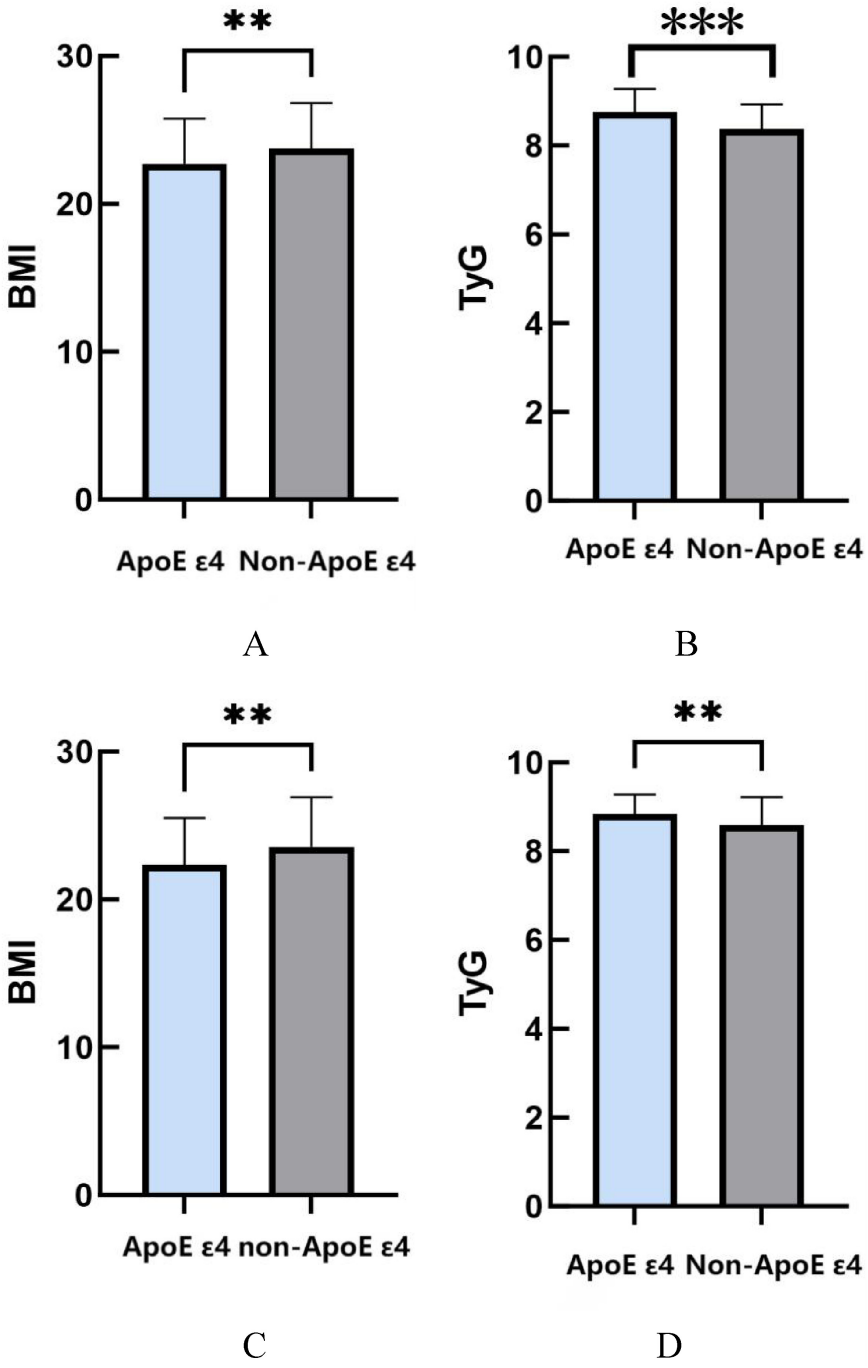
**(A)** The relationship between ApoE ε4 gene and BMI in males. **(B)** The relationship between ApoE ε4 gene and TyG in males. **(C)** The relationship between ApoE ε4 gene and BMI in females. **(D)** The relationship between ApoE ε4 gene and TyG in females. *P < 0.05; **P < 0.01; ***P < 0.001.

Stratified analyzes by sex revealed distinct metabolic patterns associated with ApoE ε4 carrier status. In male participants, ApoE ε4 carriers showed significantly lower BMI values compared to non-carriers (*P* = 0.0064), while demonstrating elevated TyG indices (*P* < 0.001). Notably, female ApoE ε4 carriers exhibited analogous metabolic patterns, with reduced BMI measurements (*P* = 0.0054) and concurrently higher TyG indices relative to their non-carrier counterparts (*P* = 0.0015) (Figure 2).

### 3.4 Age-stratified analysis of ApoE genotype and metabolic correlates

**FIGURE 3:**
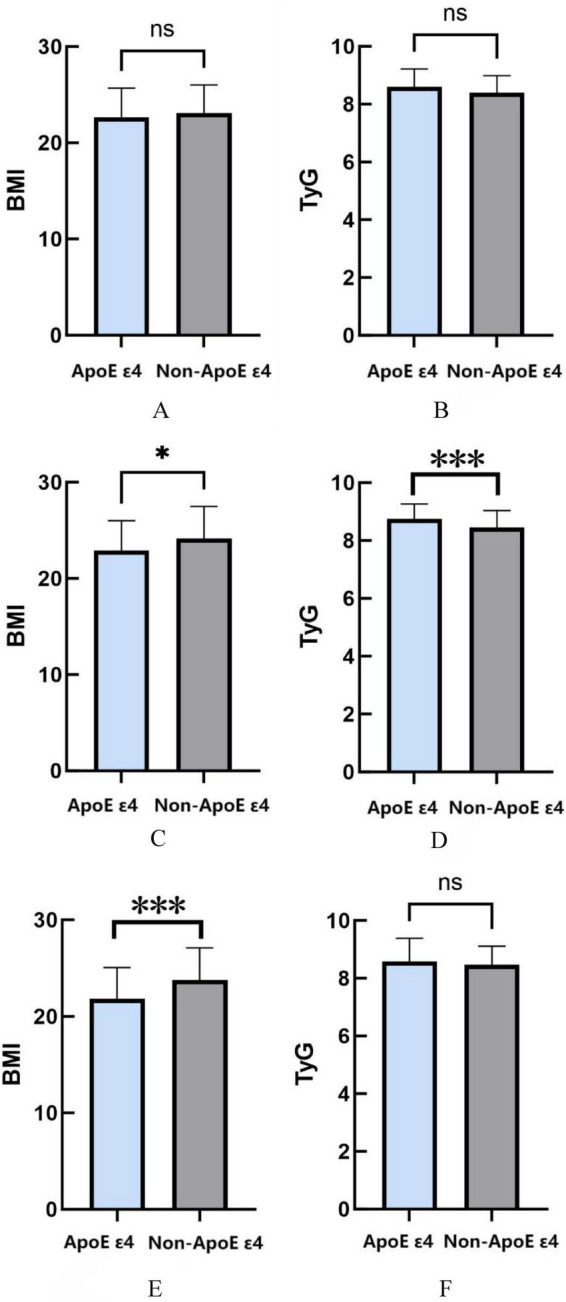
**(A)** The relationship between ApoE ε4 gene and BMI in age ≤ 65. **(B)** The relationship between ApoE ε4 gene and TyG in age ≤ 65. **(C)** the relationship between ApoE ε4 gene and BMI in age 66–74. **(D)** The relationship between ApoE ε4 gene and TyG in age 66–74. **(E)** The relationship between ApoE ε4 gene and BMI in age ≥ 75. **(F)** The relationship between ApoE ε4 gene and TyG in age 66–74. *P < 0.05; **P < 0.01; ***P < 0.001.

This study conducted age-stratified analyses to investigate associations between ApoE ε4 carrier status and metabolic parameters. In AD patients aged ≤ 65 years, no significant associations were observed between ApoE genotype and BMI (*P* = 0.305) or TyG index (*P* = 0.055). However, among participants aged 66–74 years, ApoE ε4 carriers displayed significantly reduced BMI values compared with non-carriers (*P* = 0.0122), coupled with elevated TyG indices (*P* = 0.0006). In the ≥ 75 years cohort, while TyG index showed no genotype-related differences (*P* = 0.1342), ApoE ε4 carriers maintained significantly lower BMI measurements relative to non-carriers (*P* = 0.0002) (Figure 3).

### 3.5 Cognitive-stratified associations between ApoE genotype and metabolic markers

**FIGURE 4:**
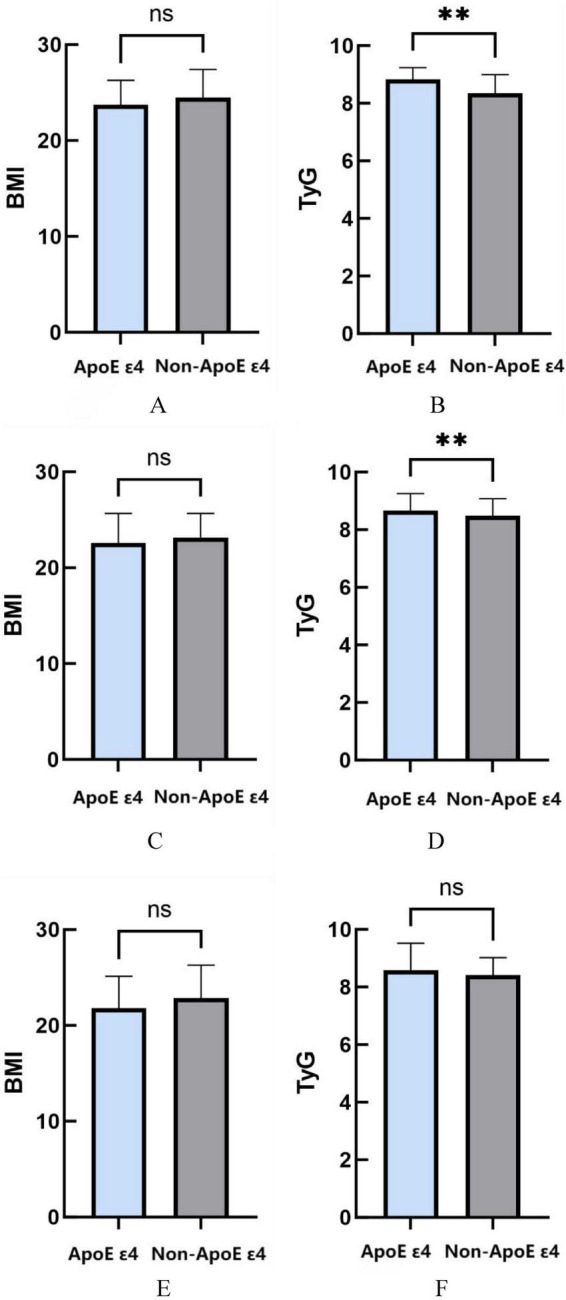
**(A)** The relationship between ApoE ε4 gene and BMI in MMSE > 20. **(B)** The relationship between ApoE ε4 gene and TyG in MMSE > 20. **(C)** The relationship between ApoE ε4 gene and BMI in MMSE 10–20. **(D)** The relationship between ApoE ε4 gene and TyG in MMSE 10–20. **(E)** The relationship between ApoE ε4 gene and BMI in MMSE < 10. **(F)** The relationship between ApoE ε4 gene and TyG in MMSE < 10. *P < 0.05; **P < 0.01; ***P < 0.001.

This study employed MMSE score stratification to analyze ApoE ε4-related metabolic variations across cognitive stages. In mild cognitive impairment (MMSE score > 20), ApoE ε4 carriers demonstrated comparable BMI (*P* = 0.286) but significantly elevated TyG indices relative to non-ApoE ε4 carriers (*P* = 0.0036). Among moderate cognitive impairment cases (MMSE 10–20), while no genotype-BMI association emerged (*P* = 0.115), ApoE ε4 carriers persistently exhibited heightened TyG levels (*P* = 0.009). Notably, severe cognitive impairment subjects (MMSE < 10) showed no significant ApoE genotype correlations with either TyG index (*P* = 0.325) or BMI measurements (*P* = 0.123) (Figure 4).

### 3.6 Mediation analysis of ApoE ε4 effects on cognitive function via metabolic pathways (TyG/BMI)

**FIGURE 5:**
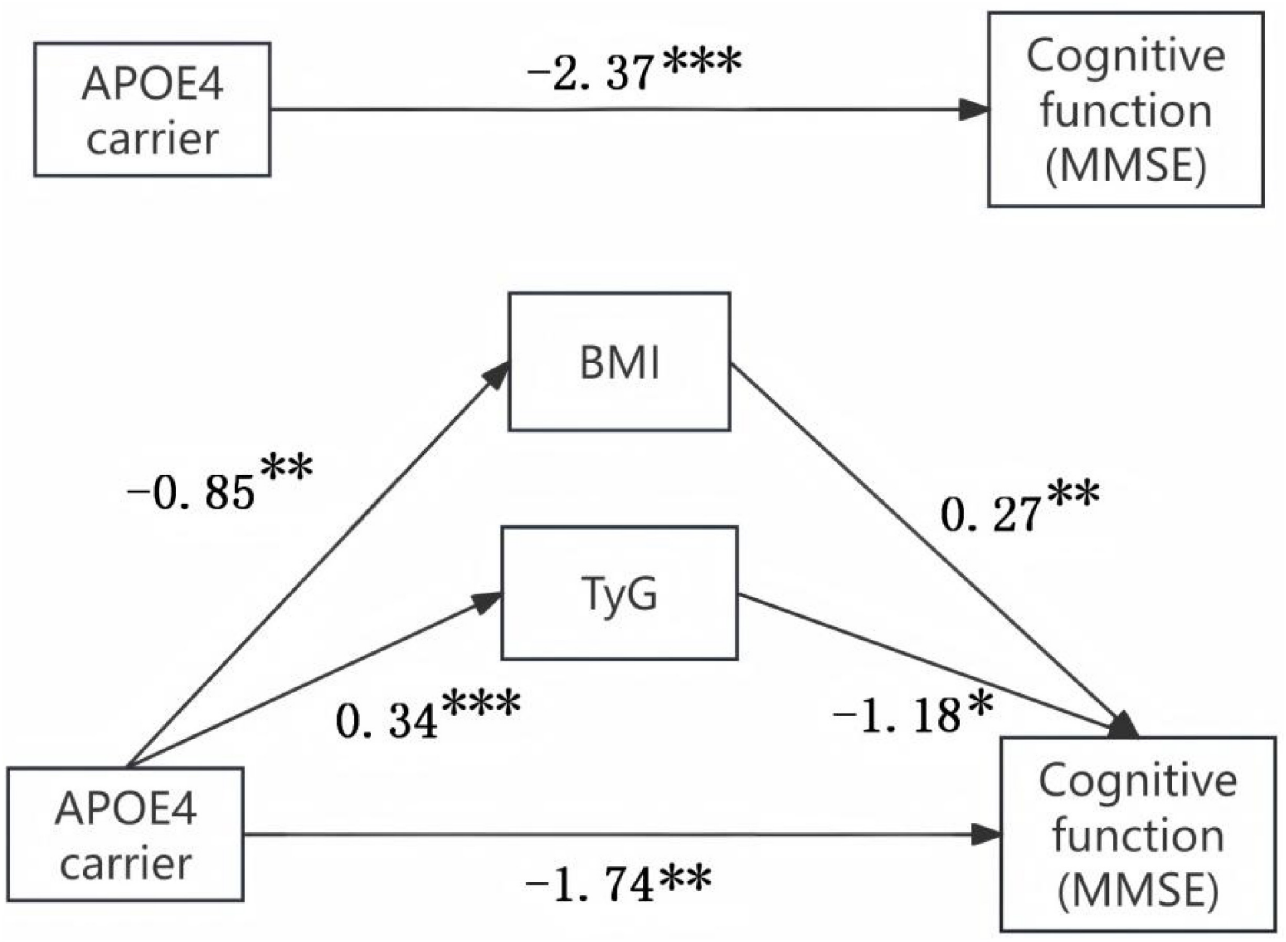
The mediation effect of ApoE ε4 gene on cognitive function through metabolic pathway. *P < 0.05; **P < 0.01; ***P < 0.001.

To investigate potential metabolic mediation pathways linking ApoE ε4 carrier status to cognitive impairment, the SPSS macro program Process Model 4 was employed to assess the mediating effect. Additionally, Hayes’ Bootstrap method was utilized to validate and analyze the mediating roles of TyG and BMI in the relationship between the ApoE ε4 and cognitive function. As detailed in Figure 5 and Table 2, the upper and lower bounds of the 95% bias-corrected confidence interval for the mediating effect of the ApoE ε4 on cognitive function, TyG, and BMI did not include zero. These findings indicate that the ApoE ε4 exerts a direct adverse effect on cognitive function and an indirect adverse effect through reduced BMI and elevated TyG. The direct effect (1.74) and the mediating effects (0.23 and 0.40) contribute 73.4, 9.7, and 16.9% to the total effect, respectively.

**TABLE 2:** The effect of ApoE ε4 gene on cognitive function through metabolic pathway.

Varianta	Effect	se	LLCI	ULCI	Effect size ratio
Total effect	−2.37	0.63	−3.60	−1.13	
Direct effect	−1.74	0.66	−3.03	−0.44	73.4%
Indirect effect 1 (BMI)	−0.23	0.13	−0.53	−0.02	9.7%
Indirect effect 2 (TyG)	−0.40	0.21	0.86	0.01	16.9%

## 4 Discussion

The research team has conducted a large-scale cross-sectional study on AD patients in northern China, exploring the link between the ApoE ε4 and metabolic factors (BMI and TyG index) and their joint impact on cognitive function. As is known to all, AD patients are often accompanied by insulin resistance, and this study also obtained similar results (Faqih et al., 2021). Results show that ApoE ε4 carriers in the AD population have significantly lower BMI and higher TyG index than non-carriers, a pattern consistent across gender. Age-stratified analyzes reveal the most significant BMI and TyG associations with ApoE ε4 in patients aged 66–74, while only BMI shows a strong link in those aged 75 and above. In terms of cognitive function levels, as measured by MMSE scores, ApoE ε4 carriers with mild to moderate cognitive impairment (MMSE > 20 and MMSE 10–20) exhibit a significantly higher TyG index than non-carriers, with no such difference observed in severe cases (MMSE < 10). Furthermore, mediation analysis suggests that ApoE ε4 may exacerbate cognitive decline indirectly by lowering BMI and raising the TyG index, accounting for 26.6% of the total effect.

As the core genetic risk factor for AD, the ApoE ε4 not only directly contributes to the central pathological mechanisms of AD, such as amyloid-beta (Aβ) deposition and neuroinflammation, but is also strongly associated with peripheral metabolic disorders. Our findings demonstrate significantly lower BMI (*P* < 0.001) and elevated TyG indices (*P* < 0.001) in ApoE ε4 carriers vs. non-ApoE ε4 carriers, suggesting ApoE ε4-mediated metabolic reprogramming involving energy homeostasis and IR contributes to AD progression.

The observed BMI reduction in ApoE ε4 carriers may reflect lipid metabolic dysregulation and cerebral bioenergetic insufficiency (Li and Liu, 2014). By disrupting lipoprotein metabolism, ApoE ε4 contributes to impaired peripheral lipid transport and disrupted lipid homeostasis in the brain. This mechanism may result in reduced fat storage and enhanced energy expenditure, ultimately leading to a lower BMI (Kypreos et al., 2009). In addition, research has demonstrated that defects in brain glucose uptake occur in the early stages of AD. Impaired brain glucose metabolism in ApoE ε4 carriers may exacerbate weight loss by promoting compensatory peripheral fat mobilization (Cunnane et al., 2016). Complementary evidence from Zhao et al., reveals ApoE ε4-mediated impairment of insulin signaling responsiveness—HDL cholesterol-induced peripheral IR synergizes with ApoE ε4 to disrupt cerebral insulin transduction (Zhao et al., 2017). Previous research has demonstrated that ApoE ε4 not only facilitates lipid accumulation in hepatocytes, contributing to fatty liver disease, but also suppresses hepatic insulin receptor signaling. These effects result in increased hepatic glucose production and elevated systemic glucose levels (Almeida et al., 2024; El-Lebedy et al., 2016; Samuel et al., 2010). In addition, ApoE ε4 may also activate the sympathetic nervous system or the hypothalamic-pituitary-adrenal (HPA) axis by impairing hypothalamic function or compromising blood-brain barrier integrity. Chronic stress elevates cortisol and epinephrine levels, stimulates lipolysis and free fatty acid release, and exacerbates peripheral insulin resistance (Martens et al., 2022; Montagne et al., 2020).

Notably, our study found that the significant association between BMI and ApoE ε4 persisted in patients aged ≥ 75 years, while the association between TyG and ApoE ε4 was lost, potentially due to the confounding effects of late-life metabolic changes resulting from disease progression (e.g., increased catabolism) or comorbidities (e.g., malnutrition). This finding aligns with the hypothesis that weight loss in late life may serve as a predictor of neurodegeneration, as observed in the context of the “obesity paradox” (Joo et al., 2018).

In addition, the TyG difference was pronounced in the mild-to-moderate stages of cognitive impairment (MMSE > 20 and MMSE 10–20) but became insignificant in the severe stage (MMSE < 10). This may be attributed to the fact that AD patients often experience systemic metabolic failure, such as malnutrition, muscle atrophy, and multiple organ dysfunction, in the advanced stages of the disease. Irrespective of ApoE genotype, metabolic indicators like TyG tend to exhibit persistent abnormalities with reduced fluctuations, thereby diminishing the distinctions between genotypes (Arnold et al., 2018). Furthermore, as the disease advances to the severe stage, extensive neuronal loss, amyloid-beta (Aβ) deposition, and tau pathology become predominant factors. Consequently, the contribution of metabolic abnormalities to cognitive decline may be overshadowed, leading to genotype-related TyG differences becoming statistically non-significant (de la Monte and Wands, 2008).

Metabolic disorders can exacerbate cognitive impairment. Cunnane et al. (2016) found that in AD patients, insulin resistance (IR)-induced hyperinsulinemia and hyperglycemia, combined with the effects of ApoE ε4, accelerates the formation of neurotic plaques (NPs). IR may also directly influence AD pathology through its interaction with amyloid-beta (Aβ) peptides. One study demonstrated a positive correlation between peripheral IR and brain Aβ deposition in the frontal and temporal lobes of AD patients. Midlife HOMA-IR was also shown to predict Aβ aggregation, with this prediction confirmed 15 years later via amyloid PET imaging (Ekblad et al., 2018; Willette et al., 2015). Laws et al. (2017) confirmed that in cognitively healthy adults, increased peripheral insulin resistance is linked to worse cognitive scores and higher cerebrospinal fluid (CSF) phosphorylated/total tau. Low BMI is significantly associated with AD-related cognitive impairment. Epidemiological studies (Grau-Rivera et al., 2021) indicate midlife or late-life low BMI may increase AD risk due to reduced nutritional status or insufficient metabolic reserves. Notably, weight loss can be an early AD biomarker, with BMI declines occurring before diagnosis. This may result from hippocampal atrophy, reduced olfactory function leading to decreased appetite, or abnormal central metabolic regulation (Grau-Rivera et al., 2021). Previous studies indicate that low BMI is linked to reduced leptin levels. This reduction impairs leptin’s ability to suppress Aβ deposition and boost hippocampal synaptic plasticity (Lieb et al., 2009). Moreover, undernutrition increases neuronal oxidative damage, and sarcopenia-induced brain-derived neurotrophic factor (BDNF) reduction further harms cognitive function (Burns et al., 2010). However, some research suggests a U-shaped relationship between BMI and AD risk, with both low and high BMI being risk factors. Also, low BMI might indicate preclinical AD pathophysiology rather than being a direct cause (Deng et al., 2022). Longitudinal studies with biomarkers like Aβ-PET and serum leptin are needed to clarify causality and guide nutritional intervention strategies.

Our study quantified the metabolic pathway’s role in ApoE genotypes’ impact on cognitive function in AD, accounting for 26.6% of the total effect. Consistent with prior studies, Seth Stoykovich et al. found that in ApoE ε4 carriers, insulin resistance (elevated TyG index) correlates significantly with memory and executive function deficits. Notably, TyG’s mediating effect (16.9%) surpasses BMI’s (9.7%), highlighting insulin resistance as a more central pathological mechanism (Cunnane et al., 2016). This aligns with the theory of AD as “type 3 diabetes” (Peng et al., 2024). Meng et al. (2025) showed that insulin resistance drives neuronal metabolic reprogramming in AD progression and emphasized that enhancing insulin sensitivity could be a viable therapeutic strategy. Thus, metabolic regulation plays a crucial role in translating ApoE genetic risk into cognitive impairment, offering new quantitative insights into AD’s gene-metabolism-cognition relationship.

Our study has several limitations: (1) Its cross-sectional design precludes causal inferences. (2) It lacks CSF biomarker measurements. (3) It doesn’t account for metabolic mediators like inflammatory cytokines [interleukin-6 (IL-6), C-reactive protein (CRP)] and adipokines (leptin, adiponectin), leaving residual mediating effects unexplained. (4) It doesn’t control for confounders such as diet and physical activity. Future research should focus on longitudinal metabolic tracking and brain PET metabolic imaging. This would help explore personalized metabolic interventions guided by ApoE genotypes.

## 5 Conclusion

This study demonstrates that ApoE ε4 carriers within the AD population exhibit significant peripheral metabolic homeostasis imbalance, characterized by decreased BMI and elevated TyG index, suggesting concurrent lipid metabolism dysregulation and insulin resistance. Furthermore, the ApoE ε4 may exacerbate AD-related cognitive impairment through metabolic pathways characterized by low BMI and elevated TyG index, with particularly pronounced effects observed in individuals aged 66–74 years and those with mild-to-moderate cognitive impairment. These findings provide crucial scientific evidence for precision prevention strategies in AD: early metabolic interventions targeting ApoE ε4 carriers (including dynamic monitoring of TyG index, nutritional status optimization, and insulin sensitivity enhancement) may represent effective approaches for delaying disease progression. Future research should integrate multi-omics data with metabolism-targeted clinical trials to elucidate the molecular mechanisms underlying metabolic regulation and validate their potential value in primary AD prevention.

## Data Availability

The original contributions presented in this study are included in this article/supplementary material, further inquiries can be directed to the corresponding authors.
